# Using natural language from a smartphone pregnancy app to identify maternal depression

**DOI:** 10.21203/rs.3.rs-2583296/v1

**Published:** 2023-02-21

**Authors:** Tamar Krishnamurti, Kristen Allen, Laila Hayani, Samantha Rodriguez, Scott Rothenberger, Eydie Moses-Kolko, Hyagriv Simhan

**Affiliations:** University of Pittsburgh School of Medicine; Allegheny County Department Of Human Services; Naima Health LLC; University of Pittsburgh School of Medicine; UPMC Western Psychiatric Hospital; University of Pittsburgh School of Medicine

**Keywords:** Depression, pregnancy, mhealth, digital health, women’s health, risk prediction, natural language processing, machine learning

## Abstract

Depression is highly prevalent in pregnancy, yet it often goes undiagnosed and untreated. Language can be an indicator of psychological well-being. This longitudinal, observational cohort study of 1,274 pregnancies examined written language shared in a prenatal smartphone app. Natural language feature of text entered in the app (e.g. in a journaling feature) throughout the course of participants’ pregnancies were used to model subsequent depression symptoms. Language features were predictive of incident depression symptoms in a 30-day window (AUROC = 0.72) and offer insights into topics most salient in the writing of individuals experiencing those symptoms. When natural language inputs were combined with self-reported current mood, a stronger predictive model was produced (AUROC = 0.84). Pregnancy apps are a promising way to illuminate experiences contributing to depression symptoms. Even sparse language and simple patient-reports collected directly from these tools may support earlier, more nuanced depression symptom identification.

## Introduction

Perinatal depression has significant impacts on both maternal and infant wellbeing. Maternal depression is associated with adverse birth outcomes, including low birth weight and preterm birth^1–2^ as well as delayed infant development^3–8^. Maternal suicide accounts for as much as 20% of postpartum mortality^9–10^. During pregnancy, approximately 15% of women report experiencing depression, making it one of the most common co-occurring health conditions in pregnancy^11^. True rates of maternal depression may be even higher due to disclosure barriers of social stigma and limited physician time to address mental health during prenatal care^12^. While screening instruments for perinatal depression have been recommended (e.g., Edinburgh Postnatal Depression Scale^13^ and PHQ-9/PHQ-2^14^), they are not consistently administered at routine prenatal care, worsening the problem of adequate detection. Moreover, while these metrics identify likelihood of a diagnosis of major depression, they offer limited insight into the underlying issues triggering or exacerbating depressed mood.

Digital health applications are now prevalent in almost every aspect of healthcare delivery in the United States^15^. Pregnancy is no exception, with more mobile health apps available for use during pregnancy than for any other clinical domain^16^. While many of these tools are directly marketed to consumers, others are designed by or delivered directly from healthcare systems and may serve as a useful form of ongoing connection to a healthcare provider in between routine prenatal visits^17^. Many individuals report the desire to use a smartphone to disclose and receive feedback about personal or sensitive health information^18^. Therefore, smartphone apps may also offer a way for pregnant people to disclose stigmatized information more easily^19^.

Natural language serves as an indicator of mental health status. The words we select and the sentiment of our statements offer a window into our wellbeing^20^. Moreover, the themes in our narratives may identify the foci of our rumination. Recent promising natural language processing (NLP) models have included information extracted from large-scale datasets in conjunction with more domain-specialized language guided by practitioner expertise^21–25^. Zhang and colleagues reviewed studies specifically detecting mental illness using NLP methods and found that the vast majority applied these methods to text that had been extracted from large social media forums or electronic health records^26^. These NLP models of prediction generally seek out extensive datasets because the black box approaches they employ require them to train large numbers of parameters. A much smaller number of the studies they reviewed – 2% – used an individual’s own narrative writing to identify depression. However, even limited amounts of narrative text may be useful in identifying an individual at risk of poor mental health^27^. Thus, narrative language provided by patients through pregnancy apps may offer an additional opportunity for healthcare providers to identify maternal depression risk while gaining a better understanding of the underlying nature of an individual patient’s stressors.

**Figure 1:**
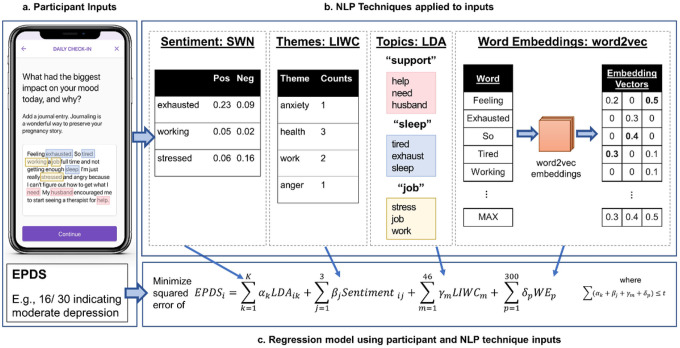
NLP techniques. (a) An artificial example of open-ended text that might be entered into the app is shown; (b) Four NLP methods are used to extract language features from the text; (c) The combination of language features is put into the regression model to obtain a predicted EPDS score. This figure has been modified from a figure previously published by the authors^52^.

Here, we collected first-person narrative text entries as well as depression symptom scores using a prenatal care app prescribed by obstetrical care providers to their patients for use during pregnancy. We then used these data to predict depression symptom scores from features of natural language, employing four distinct NLP approaches. Figure 1 details the process by which we generated 4 sets of unique language feature scores that can be entered as inputs into a single regularized linear regression predicting depression symptom scores. Sentiment analysis tools (here we used SentiWordNet) estimate positive or negative valence in words and clarify the writer’s attitude about their subject^28^. The Linguistic Inquiry and Word Count dictionary (LIWC), where expert insight seeded psychologically-informed themes, has shown utility in identifying pre-specified topics associated with mental health issues^21, 24, 29, 30^. Topic modeling can elucidate emergent themes particular to specific domains of writing^31^ that may not be present in more generalized NLP datasets developed on news articles and historical texts. Word2vec features provide a representation of words’ syntactic and semantic features based on the context of their use in vast bodies of pre-training text^32^.

The goal of this study was to determine if (a) the type of digital support routinely offered to patients as part of prenatal care could be used to identify pregnancy depression symptoms from patient-entered natural language and (b) if those natural language features could provide insight into the most salient themes associated with depression symptoms during pregnancy. This study was pre-registered on the Open Science Framework at https://osf.io/tydfh.

## Results

### Study participants

**Figure 2:**
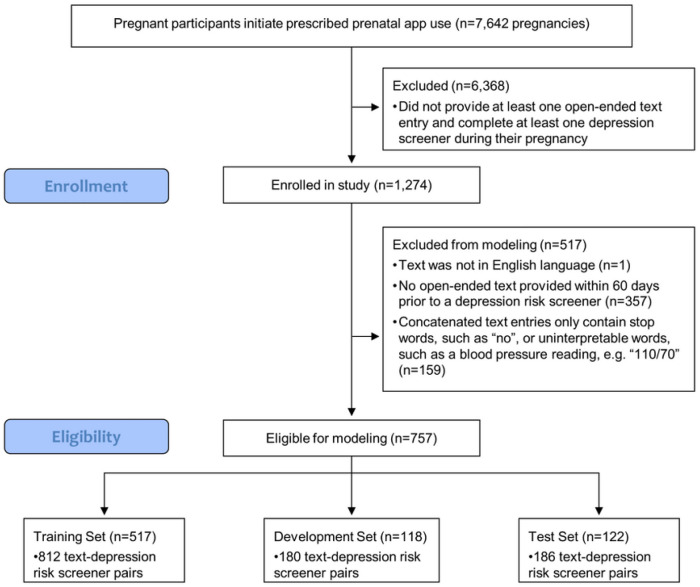
STROBE flow diagram. Flowchart of enrollment in study, eligibility criteria for modeling, and split into training, development, and testing groups.

During the study period of September 2019 - October 2022, the MyHealthyPregnancy app was used by 7,455 individuals who had been prescribed the app as part of their routine prenatal care and agreed to share their deidentified data for scientific research. While engaging with the larger set of pregnancy monitoring and education features included in the app, individuals experiencing a total of 1,274 pregnancies (a small number used the tool for more than one pregnancy) provided at least one open-ended text entry and voluntarily completed at least one 10-item Edinburgh Postnatal Depression Scale (EPDS) to self-report depression symptoms in the app. This subset of app users was then enrolled in the study and randomly divided into a test set, development set, and training set with a respective split of 15%, 15% and 70% of participants (Fig. 2). Of these, 59.42% (N = 757) provided enough language in open-ended text entries for meaningful feature extraction within the 60-day period preceding an EPDS score. The final split was 122 participants (16.1%) in the test set, 118 participants (15.5%) in the development set, and 517 participants (68.3%) in the training set.

Table 1 displays the baseline demographic categories and relevant clinical history, which were provided by participants upon initiating app use and have been shown in prior literature to predict depression in pregnancy^33^. Most participants identified as non-Hispanic White/Caucasians and were partnered. The mean age of participants was 29.8 years (SD = 5.4 years). Most had a college education or higher. The majority reported a family household income of at least $50,000 annually. The demographic characteristics of those included in the study were largely reflective of the greater population of MyHealthyPregnancy app users, with few significant differences between app users enrolled in the study and app users who were not enrolled. However, participants who were enrolled were more likely to have a household income of less than $50,000/year and were more likely to report histories of depression and/or anxiety (Supplementary Table 1).

### Depression screening

Participants completed an EPDS on average 2.5 (SD = 2.3) times over the course of their pregnancy with the median time of first completion being the 11th week of pregnancy. Among all averaged EPDS scores that were eligible for modeling, 55.0% indicated no depression symptoms (score of 0–6), 34.6% indicated mild depression symptoms (score of 7–13), 7.7% indicated moderate depression symptoms (score of 14–19), and 1.9% indicated severe depression symptoms (score of 20–30). These EPDS scoring cut-points reflect those used by the UPMC healthcare system and others. One percent (17/1496) of EPDS scores included in modeling indicated suicidal ideation at the time of the quiz.

### Open-ended text entries

Participants provided open-ended text through different features of the tool, including responses to specific prompts and a pregnancy journaling section of the app. Text entries provided in close temporal proximity (e.g., a journal entry on Day 1 and an open-ended response on Day 10) were concatenated, producing 1 to 10 unique open-ended text entries per participant, with an average of 1.56 entries. Text entries were an average length of 53 (SD = 115) words. Lengthier entries often included descriptions of multiple experiences, such as physical symptoms, notes from medical appointments, and narratives of personal events. For example, a participant might share writing, such as,

I’m obsessing, getting the baby’s room ready. There’s a ton of stuff out there but not sure what’s immediately necessary. Also had a migraine this morning (took some meds) and issues with food aversions this week. Having some mood swings too, it comes and goes throughout the day but it’s alright for now.

Shorter entries were typically explanations in response to open-ended prompts (e.g., “*What had the biggest impact on your mood today, and why?*” with a sample response of “*Good mood today b/c of ultrasound*”) rather than unprompted entries.

### Self-reported mood on a Likert scale

Participants could also self-report mood through the app as frequently as once a day on a 5-point Likert scale. Participants responded to a prompt of “How is your mood today?”; reporting daily mood scores ranging from 1 (“Very Poor”) to 5 (“Very good”). Among the text entries eligible for modeling, 92% of entries were paired with at least one co-occurring report of mood. with a mean mood across participant responses of 3.88. Among those who shared their current mood in the app, mood was reported an average 5.4 times in the month prior to completing an EPDS.

### Depression model using language inputs derived from text entries

In a Least Absolute Shrinkage and Selection Operator (LASSO) model (see Table 2), which included baseline demographic categories and relevant clinical history, natural language features were found to be predictive of moderate to severe prenatal depression symptoms occurring in a subsequent 30-day timeframe in the test set (AUROC = 0.72) and to a slightly lesser extent in the subsequent 60-day timeframe (AUROC = 0.69). The addition of self-reported current mood around the time of text entry noticeably increased the predictive performance of the model (AUROC = 0.84 and AUROC = 0.81 for 30-day and 60-day timeframes, respectively).

The number of NLP features retained in each model and their coefficients are shown in Table 3. The 30-day and 60-day timeframe models retained 17 and 19 natural language features, respectively. Across both timepoints, sentiment fluctuation (SentiWordNet differences in positive and negative sentiment scores), LIWC (psychologically-informed) themes, and word2vec (underlying semantic and syntactic) features all predicted depression symptoms. When self-reported mood was included, sentiment fluctuation was no longer retained in the model for either timeframe, and several LIWC themes were dropped. Additionally, when mood was included, a greater number of word2vec features were retained, with the maximum coefficient among word2vec features increasing from 0.17 to 0.26 in the 60-day timeframe. Emergent topics (from topic modeling) were not retained in any of the models.

Table 4 shows coefficients of the most predictive SentiWordNet score (sentiment fluctuation), the LIWC summary characteristics and psychological process themes, and the most predictive word2vec features that were associated with subsequent moderate to severe depression symptoms. In both the 30-day and 60-day models, sentiment fluctuation (i.e., the difference between the degree of positive and negative sentiment present in text entries) was negatively associated with subsequent depression symptoms, although the strength of the relationship between sentiment fluctuation and depression symptoms was much stronger in the 30-day time period. In both time periods, one of the most protective language features against depression symptoms was the use of first-person plural pronouns, such as *we and ours*. Language related to *want* (e.g., *wish, crave, hope*), language related to *mental health (e.g., antisocial, trauma, suicide*), and language related to space (e.g., *home, around*) were all positively associated with subsequent depression symptoms. Language related to time (e.g., *today, finally*) was negatively associated with subsequent depression symptoms.

When participants’ current mood was added to the 30-day model, only the LIWC theme of *mental health* was retained, though with a reduced coefficient, and language related to *pregnancy & reproductive health* emerged as positively associated with subsequent depression symptoms. The tone of writing was strongly associated with decreased depression symptoms in the language models in both timeframes but was no longer retained once current mood was accounted for. Word count had a small but positive association with depression symptoms in all models.

A modified text sample indicative of moderate to severe depression is as follows:

I haven’t been sleeping well, so it’s hard to get out of bed. Then it’s hard for me to cope with the stress at work. I’m so tired and I just want to be at home. My husband and I talked about how to support me more because I’m really struggling doing all this on my own. I want this fatigue to stop ASAP. Today I had an ultrasound - the heart rate was 130. I think that means ITS A BOY and I’m kind of freaking out

A modified text discussing the same topics but without depression-indicative language features is as follows:

I’ve been working a lot lately and sleep isn’t as easy as it used to be. I’m tired a lot, but my husband’s been taking care of chores in the evening to lighten the load. And today we had an ultrasound and got to see our little girl moving around!! I can’t wait to watch her grow, and I’m so happy we’re all in this together.

Lastly, several word2vec features were positively associated with depression symptoms. Word2vec feature 166, for example, was positively associated with depression symptoms at both 30-day and 60-day timepoints, whether or not mood was included in the model. Upon qualitative review of these word2vec features, it was not immediately obvious what underlying language dimension differentiates those with high scores from those with low scores. Those with the highest scores on feature 166, for example, tended to write lengthier, more detailed responses than those with the lowest scores, and often referenced interpersonal conflict (e.g., fights with partners, tension with co-workers). Given this qualitative observation, it is possible that response length is an aspect of the underlying structure driving the positive link between word2vec features and depression but does not fully explain the relationship between word2vec features and degree of depression symptoms.

## Discussion

In this observational prospective cohort study, we modeled depression symptoms using NLP outputs from sparse written text collected through a pregnancy smartphone app that was delivered to patients are part of their routine prenatal care. The model had performance matching or better than other machine learning models for maternal depression prediction, which often have been built on larger data sets with a greater number of variables^34–35^. Our results add to a new, but growing, literature indicating that even sparse language can be used to predict depression symptoms. Moreover, this study focuses on a population for whom depression can have severe consequences. By capturing language through a prenatal smartphone app, this study also lays a foundation for wider-scale remote assessment of maternal depression from patients’ everyday language.

Specifically, we find that natural language features, including tone, first-person plural pronoun use, specific topics, such as *space, mental and pregnancy-related health*, and temporal *wants*, context-derived syntactic and semantic dimensions, and word count are indicative of depression symptoms. Moreover, these features capture a unique aspect of symptom level beyond current mood or baseline demographics or clinical risk factors. The best-performing model identified incident depression in a 30-day window with mood, topics focused on mental health and pregnancy-related health issues, and syntactic/semantic features all associated with depression symptoms. Pronoun use and topics associated with depression symptoms could reflect aspects of social isolation, e.g., use of “I” rather than “we” and references to staying in or needing to be in certain physical *spaces*. Our results also shed light on the types of topics that current mood may be capturing, such as temporal desires (captured by the LIWC topic, *wants, - e.g., “wish”, “hopeful”*).

In an illustrative contrast to prior studies finding that use of the first-person singular is associated with depression^36–40^, we find that first-person plural pronoun use is negatively associated with (or protective against) depression symptoms. The use of first-person plural pronouns in our predominantly partnered sample, particularly during pregnancy, could be indicative of the strength of the existing family structure. Use of “we” rather than “I” when discussing pregnancy may indicate degree of bonding in the partnership unit or the mother-infant dyad, consistent with literature on the protective effects of social support and mother-child bonding^41^. This linguistic focus on first-person plural pronouns may indicate a protective counterpoint of social supports in opposition to the self-focus or self-criticism suggested by first-person singular pronouns that has been shown to be harmful in prior work^35, 42–43^.

Notably, the theme of *mental health* was retained as a predictive language feature in the 30-day timeframe when controlling for mood and other baseline characteristics. That those with moderate to severe depression symptoms were writing about their mental health (e.g., *psychiatrist, zoloft, trauma*) is suggestive of the writer’s existing understanding of their depression status and perhaps a sensitivity to their own ebbs and flows. Previous studies have shown that those who are depressed may find writing therapeutic^44^, while others have found that re-living events can be either therapeutic or harmful^45^. Here, when given the opportunity to share writing in a pregnancy app, individuals experiencing depression symptoms wrote about their mental health and wrote more extensively than those who were not depressed. In addition to being a tool for eliciting depression symptoms in between routine prenatal care, such tools may offer an additional opportunity for sharing, particularly if structured in a way to support therapeutic rather than harmful disclosure of experiences. To do this effectively, future work should explore the structure of digital tool-based elicitation of writing to understand which prompts and formats of writing-elicitation allow for therapeutic disclosure.

Much of our data was collected during a pandemic and through periods of mandated self-isolation with fewer in-person clinical appointments. However, even though COVID-19 was explicitly included as a novel LIWC theme in modeling, it was not retained as an indicator for depression symptoms in our modeling. This result suggests that other topics, which may be consequent to COVID-19 pandemic experiences but do not specifically reference COVID-19 precautions or symptoms, are more directly indicative of depression.

Consistent with other literature^46–48^, we find that a lack of fluctuation in a text’s sentiment is symptomatic of depression (i.e. less varied – or “flattened” – affect in language is associated with depression symptoms). We also find that high-dimensional representations of the underlying syntactic and semantic content of open-ended text, captured by word2vec features, were indicative of depression symptoms, even more so when paired with self-reported mood. While the word2vec features are not easily interpretable, these findings suggest that there is something about underlying word choice that is uniquely informative and distinct from explicitly psychologically meaningful themes. Future work should examine whether these word-based language features could be used as an automated trigger for depression screening among patients of a specific healthcare system, as has been discussed in the context of social media^49^.

Our findings should be interpreted in the context of its naturalistic, patient-led data generation. While the self-motivated collection approach translates clearly to practice, the resulting data are sparse and tend to be highly topically focused. Thus, we may not have fully captured the range of language features that could indicate depression symptoms among pregnant people in a more directed data collection structure. For example, we did not find any emergent topics associated with depression symptoms in this sample, likely due to topic models’ need for larger bodies of text. It is possible that the number and, especially, length of the written texts may not have been sufficient to expose more subtle or infrequent but meaningful themes. Future work could include manual coding of the entries to clarify more nuanced themes and experimental data collection with and without clear writing prompts.

The naturalistic structure of the study added some noise to our text. Some individuals used open-ended text opportunities to track blood pressure readings or take notes on medical appointments. How individuals used the open-ended writing opportunities in the tool is something that could be explored in future work. We find that individuals with a history of depression or anxiety tend to use the overall tool for longer and write more frequently. However, this disproportionate use of the tool by those who have a history of depression may also be a strength if prenatal apps offer an additional means of therapeutic disclosure and connection to patients who are more likely to become depressed.

To the best of our knowledge, this is one of the first prospective longitudinal studies to use natural language collection^50^ and the first focused on maternal depression symptom prediction. Incorporating language inputs enables moderate predictive ability of depressive symptoms among peripartum patients in a large academic health system. This work points to an immediate value in using digital tools for depression symptom evaluation and support between routine clinical care appointments. It also indicates the potential for future analysis of app-elicited language to trigger mental health care provision.

## Methods

### Participant enrollment

The MyHealthyPregnancy smartphone application was prescribed to patients receiving prenatal care in the UPMC healthcare system as part of a quality improvement initiative (Quality Improvement/Ethics board approval project number: 1684). Prescriptions were typically made between the 7th and 10th weeks of pregnancy, at a patient’s first prenatal visit. Upon downloading the app and creating an account, participants electronically consented to the dissemination of de-identified aggregate data for scientific development. The specific analyses presented here, collected from a longitudinal observational cohort of patients, were approved by the University of Pittsburgh’s Institutional Review Board (STUDY19100210)) There was no financial compensation provided to participants for app use.

### Data collection

Upon initiation of app use, participants completed baseline questions, including sociodemographic information regarding their race/ethnic identity, household income, education level, and relationship status. They were additionally asked about their history of diagnosed depressive and anxiety disorders. Participants could record information about their pregnancy experience on a routine (up to daily) basis using a “check-in” questionnaire that included a question about their mood that day, with response options recorded on a 5-point Likert scale.

Throughout their pregnancy, participants were able to share open-ended text in the app in several ways. First, the app contained a dedicated section for participants to voluntarily document their thoughts, feelings, symptoms, or other notes. Second, participants had opportunities to share open-ended responses to routine (weekly or monthly) questions about their current experience, covering mental and relationship wellbeing (e.g., *What had the biggest impact on your mood today, and why?*). Lastly, from April 2020 to September 2022, an app-embedded COVID symptom screener also included open-ended text questions asking users about their sources for COVID-19 information, preferred methods to prevent infection, and challenges during the pandemic (i.e., *Are you experiencing financial or other personal difficulties as a result of this pandemic?*).

The app also allowed participants to complete the Edinburgh Postnatal Depression Scale (EPDS) to monitor their mental health throughout their pregnancy. Participants were prompted on the app’s home screen once a trimester to complete the questionnaire throughout the course of the study. Starting in June 2021, an update in the app made the EPDS available to participants to complete at any time.

### Inclusion criteria

All users were eligible to participate if they had provided consent for deidentified aggregate analysis of their data for research purposes, completed an EPDS at some point during their use of the app, and entered one or more usable open-ended text entries within the 60-day window prior to providing an EPDS score. Usable text entries were defined as English-language entries that retained at least one meaningful word after concatenated text entries had been processed to remove less meaningful words. Word removal applied to text before extraction of word2vec features, LDA topics, and sentiment. This included stop-words (such as “I”, “be”, and “did”) and overly common words found in responses to open-ended text prompts (such as “today” and “yes”). Pre-processing also removed capitalization, punctuation marks, and accent marks.

### Pairing open-ended text with depression symptom scores

To ensure that open-ended text entries could be used to model mental status, text and EPDS pairings were identified for which text entries preceded self-reported EPDS scores within a fixed time. Two fixed time frames were selected. A 60-day window was chosen to reflect the DSM-5 criteria for major depressive disorder, which defines remission as 2 or more months of little to no depressive symptoms. A shorter 30-day window was chosen to reflect the timeframe often used by clinicians to identify depressive symptoms for a new depression diagnosis.

**Figure 3:**
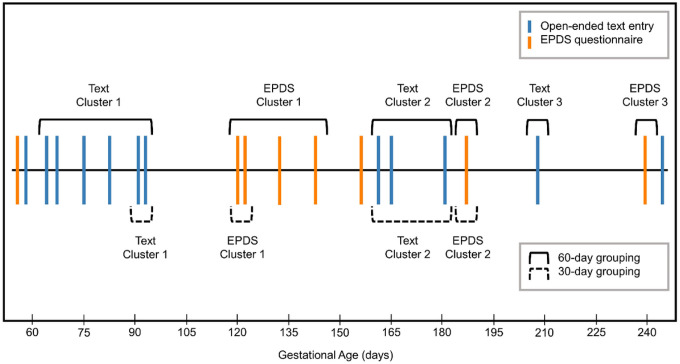
Example of pairing open-ended text entries with depression symptom assessments (EPDS) in the 30-day and 60-day timelines.

Participant data was first processed by grouping together open-ended text entries with following EPDS scores. Open-ended text entries within 60 days preceding an EPDS score were concatenated together. This concatenated text was then paired with the average of all following EPDS scores within 60 days from the last open-ended text entry in the cluster before the occurrence of a newer text entry. The same process was completed to create text and EPDS score pairings in a 30-day timeframe. With both timeframes, EPDS scores with no preceding text entries and text entries with no following EPDS scores were eliminated from the dataset. In models that included reports of mood, mood data was only included if within the same timeframe as open-ended text entries. Figure 3 shows an example of data grouped together in the 60-day and 30-day timeframe. Multiple reports of mood in the 30-day or 60-day window were averaged before use in the regression model.

### Natural language processing methods

#### Sentiment fluctuation

Text sentiment was analyzed using SentiWordNet^28^. Each word extracted from a processed text entry was stemmed and given a positive or negative score using a stemmed SentiWordNet dictionary. Words that had multiple entries within the dictionary were given the average of those scores. Positive and negative scores across an entire processed text entry were averaged to give an average positive sentiment and an average negative sentiment. Finally, overall fluctuation in sentiment was calculated by subtracting the average negative from average positive scores.

#### Themes and topics

Topic modeling was performed using Latent Dirichlet Allocation (LDA), an unsupervised machine learning method that clusters data points into a predetermined number of topics^31^. The number of LDA topics, k, was selected by iterating across five splits of the training set and evaluating the resulting topics for predictive capacity on EPDS using LASSO. The *k* between 1 and 50 leading to best performance was used to run a new, final set of topics on the full dataset. The optimal k value was found to be 5 topics. Topic models, an unsupervised method, permit us to examine domain-specific patterns that may emerge in this text by pregnant people as distinct from general usage, and from the news media text used to train word2vec, by illustrating which less-common words frequently occur together in this body of text.

The Linguistic Inquiry and Word Count dictionary (LIWC-22) was used to count the number of occurrences of 119 themes, grammatical features, and positive and negative affect within each text entry^29^. In addition to 117 LIWC-22 themes, two additional themes were manually created to capture domain-specific content. A COVID-19 theme included terminology related to the global health crisis, such as “mask”, “booster”, and “pandemic”. A second theme was created for pregnancy-specific health, which captured pregnancy terminology not fully captured in pre-existing LIWC categories. This theme included common pregnancy-related symptoms, such as “heartburn” and “contractions,” as well as words that are specific to healthcare services provided in pregnancy, e.g., “doula” or “amniocentesis.” In contrast to the other NLP methods, LIWC was used on un-processed text entries to ensure the capture of pronouns, conjunctions, and other function words.

#### Syntactic/semantic features

Word embeddings incorporate high-dimensional context-derived representations of syntactic and semantic information for each concatenated text entry. The 300-dimensional word2vec embeddings were pre-trained on word co-occurrence and proximity in 1.6 million news articles, giving similar representations to words that usually showed up in the same contexts^32^. Here, a word embedding vector was retrieved for each word in the entry, and each entry was represented by a vector of the highest value for each of the 300 features. This approach acknowledges that there is systematic regularity among word embeddings that captures meaningful information about semantic and syntactic roles^51^. While individual features are not manually interpretable, we build from the assumption that they are independently meaningful to consolidate word embeddings from each journal entry into a single summary vector.

#### Statistical analysis

EPDS scores were predicted for each data point in the data set using LASSO, a penalized linear regression model that reduces over fitting. Natural language features were standardized within the dataset prior to modeling, with a mean of 0 and a standard deviation of 1 for each feature. A five-fold cross validation was used on the training + development set to find the optimal shrinkage penalty for the LASSO regression in a range from 0.0001 to 1. The value minimizing averaged mean squared error in predictions was then used to train the LASSO on the entire training and development dataset. Continuous EPDS score predictions were used to calculate AUROC in predicting true test set scores with a threshold of > 13.

LASSO regression was run in the 60-day and 30-day timeframe with NLP features and demographic information. This included the participant’s age at the start of their pregnancy, their race/ethnicity (White, Black, Hispanic/Latinx, Asian, or other), a binary variable for having a household income of $50,000 or more, a binary variable for having an associate degree or higher, self-reported history of depression, and self-reported history of anxiety. Secondary LASSO regressions were run in the 60-day and 30-day timeframe among the same data points that included demographic information only to serve as a comparison point.

Two exploratory analyses explored the importance of mood variation and the presence of bothersome physical symptoms by combining demographic information and language features with minimum mood, maximum mood, average mood, and a binary variable for the reporting of physical symptoms in the 60-day and 30-day timeframes.

## Figures and Tables

**Table 1:** Demographics of study participants.

Characteristic	Enrollees (n = 1,274)
Age, mean (SD)	29.8 (SD = 5.4)
Income, US dollars, thousands	
<15	155 (12.2%)
15 to <50	321 (25.2%)
50 to 100	427 (33.5%)
>100	341 (26.8%)
Missing or preferred not to respond	30 (2.3%)
Race and Ethnicity	
White/Caucasian	1010 (79.3%)
Black or African American	137 (10.8%)
Hispanic/Latinx	26 (2%)
Asian	42 (3.3%)
Another ethnicity	52 (4.1%)
Missing or preferred not to respond	7 (0.5%)
Educational Level	
<High school	32 (2.5%)
High school or GED	371 (29.1%)
Associate degree	146 (11.5%)
Bachelor degree	364 (28.6%)
Postgraduate degree	353 (27.7%)
Missing or preferred not to respond	8 (0.6%)
Relationship Status	
Partnered	1207 (94.7%)
Not partnered	64 (5%)
Missing or preferred not to respond	3 (0.2%)
Medical History	
History of depression	308 (24.2%)
History of anxiety	384 (30.1%)
Parity	
Nulliparous	628 (49.3%)
Multiparous	646 (50.7%)

**Table 2:** Summary of LASSO results.

Features	Timeframe	No. of entries in the model	Training + development set AUROC	Test set AUROC	Test set R^2^
Baseline risk factors only	30-day	1077	0.76	0.67	0.06
	60-day	1152	0.76	0.67	0.08
Baseline risk + NLP features	30-day	1077	0.79	0.72	0.09
	60-day	1152	0.79	0.69	0.10
Baseline risk, NLP features, Mood + Symptoms	30-day	988	0.89	0.84	0.23
	60-day	1055	0.88	0.81	0.23

**Table 3:** Summary of language feature coefficients in each model.

Model	Features							
	*SentiWordNet*		*Topic Modeling*		*LIWC*		*Word2vec*	
	Number	Range	Number	Range	Number	Range	Number	Range
Baseline risk + NLP features	1	−0.12	0	-	8	[−0.31, 0.19]	8	[0.00, 0.13]
	2	[−0.06, 0.07]	0	-	9	[−0.32, 0.19]	8	[0.05, 0.17]
Baseline risk, NLP features, Mood + Symptoms	0	N/A	0	-	6	[−0.01, 0.14]	12	[0.01, 0.14]
	0	N/A	0	-	1	0.05	9	[0.01, 0.26]

**Table 4:** Language features that were found to be most predictive in the best-performing models.

Language Features	Description or exemplar words	Models with Baseline risk + NLP features		Models with Baseline risk, NLP features, Mood + Symptoms	
		30-day	60-day	30-day	60-day
SentiWordNet					
Sentiment fluctuation	Positive minus negative sentiment	−0.12	−0.06	-	-
LIWC					
Clout	Language that indicates social status	−0.03	-	-	-
Tone	Sum of positive and negative tone	−0.31	−0.33	-	-
First person plural	*We, our us, let’s, etc*.	−0.17	−0.26	-	-
Mental Health	*Depressed, suicidal, trauma, psychiatrist*, etc	0.11	0.08	0.06	-
Pregnancy/ Reproductive Health	*Pregnant, pregnancy sex, cervix*, etc.	-	0.01	0.14	-
Want(s)	*Wish, wanna, hopeful, craving*, etc.	0.19	0.19	-	-
Space	*In, out, here, home*, etc.	0.09	0.06	-	-
Time	*Day week, ever, now*, etc.	−0.01	−0.08	-	-
Perception	*Feel, ear see, going*, etc.	-	0.01	-	-
Third person singular pronouns	*She, he, her, him, etc*.	-	-	0.02	-
Impersonal pronouns	*It, that, everything, everyone*, etc.	-	-	−0.01	-
Causation	*Because, how, make, results*, etc.	-	-	0.02	-
Word Count	Number of words and emoticons	0.03	0.06	0.04	0.05
Word2vec features					
Word2vec #166		0.13	0.17	0.09	0.11
Word2vec #127		-	0.12	0.14	0.26
Word2vec #159		0.09	0.10	-	-
Word2vec #167		-	-	0.12	-
Word2vec #298		0.10	0.05	-	-

## Data Availability

Original identifiable data are not publicly available to protect patient privacy and due to the terms and conditions of app data use. De-identified natural language feature data will be available in the National Institute of Mental Health Data Archives.

## References

[1] Grote N. K. A meta-analysis of depression during pregnancy and the risk of preterm birth, low birth weight, and intrauterine growth restriction. Archives of General Psychiatry 67, 1012 (2010). 20921117 10.1001/archgenpsychiatry.2010.111 PMC3025772

[2] Chung T. K. H., Lau T. K., Yip A. S. K., Chiu H. F. K. & Lee D. T. S. Antepartum depressive symptomatology is associated with adverse obstetric and neonatal outcomes. Psychosomatic Medicine 63, 830–834 (2001). 11573032 10.1097/00006842-200109000-00017

[3] Cummings E. M., Schermerhorn A. C., Keller P. S. & Davies P. T. Parental depressive symptoms, children’s representations of family relationships, and child adjustment. Social Development 17, 278–305 (2008).

[4] Elgar F. J., Mills R. S., McGrath P. J., Waschbusch D. A. & Brownridge D. A. Maternal and paternal depressive symptoms and child maladjustment: The mediating role of parental behavior. Journal of Abnormal Child Psychology 35, 943–955 (2007). 17577659 10.1007/s10802-007-9145-0

[5] Grace S. L., Evindar A. & Stewart D. E. The effect of postpartum depression on child cognitive development and behavior: A review and critical analysis of the literature. Archives of Women’s Mental Health 6, 263–274 (2003). 10.1007/s00737-003-0024-6 14628179

[6] Lim J. H., Wood B. L. & Miller B. D. Maternal depression and parenting in relation to child internalizing symptoms and asthma disease activity. Journal of Family Psychology 22, 264–273 (2008). 18410213 10.1037/0893-3200.22.2.264

[7] Cummings E. M. & Davies P. T. Maternal depression and child development. Journal of Child Psychology and Psychiatry 35, 73–122 (1994). 8163630 10.1111/j.1469-7610.1994.tb01133.x

[8] Lovejoy M. C., Graczyk P. A., O’Hare E. & Neuman G. Maternal depression and parenting behavior. Clinical Psychology Review 20, 561–592 (2000). 10860167 10.1016/s0272-7358(98)00100-7

[9] Lindahl V., Pearson J. L. & Colpe L. Prevalence of suicidality during pregnancy and the postpartum. Archives of Women’s Mental Health 8, 77–87 (2005). 10.1007/s00737-005-0080-1 15883651

[10] Campbell J., Matoff-Stepp S., Velez M. L., Cox H. H. & Laughon K. Pregnancy-associated deaths from homicide, suicide, and drug overdose: Review of research and the intersection with intimate partner violence. Journal of Women’s Health 30, 236–244 (2021). 10.1089/jwh.2020.8875 PMC8020563 33295844

[11] Dietz P. M. Clinically identified maternal depression before, during, and after pregnancies ending in live births. American Journal of Psychiatry 164, 1515–1520 (2007). 17898342 10.1176/appi.ajp.2007.06111893

[12] Dennis C.-L. & Chung-Lee L. Postpartum depression help-seeking barriers and maternal treatment preferences: A qualitative systematic review. Birth 33, 323–331 (2006). 17150072 10.1111/j.1523-536X.2006.00130.x

[13] Cox J. L., Holden J. M. & Sagovsky R. Detection of postnatal depression. British Journal of Psychiatry 150, 782–786 (1987). 10.1192/bjp.150.6.782 3651732

[14] Kroenke K. & Spitzer R. L. The PHQ-9: A new depression diagnostic and severity measure. Psychiatric Annals 32, 509–515 (2002).

[15] Abernethy A. The promise of Digital Health: Then, now, and the future. NAM Perspectives 6, (2022). 10.31478/202206e PMC9499383 36177208

[16] Hughson J. A. P., Daly J. O., Woodward-Kron R., Hajek J. & Story D. The rise of pregnancy apps and the implications for culturally and linguistically diverse women: Narrative review. JMIR mHealth and uHealth 6, (2018). 10.2196/mhealth.9119 PMC6269626 30446483

[17] de Mooij M. J. Ob nest: Reimagining low-risk prenatal care. Mayo Clinic Proceedings 93, 458–466 (2018). 29545005 10.1016/j.mayocp.2018.01.022

[18] Krebs P. & Duncan D. T. Health app use among US mobile phone owners: A national survey. JMIR mHealth and uHealth 3, (2015). 10.2196/mhealth.4924 PMC4704953 26537656

[19] Krishnamurti T. Mobile remote monitoring of intimate partner violence among pregnant patients during the COVID-19 shelter-in-place order: Quality Improvement Pilot Study. Journal of Medical Internet Research 23, (2021). 10.2196/22790 PMC7899202 33605898

[20] Andreasen N. J. Linguistic analysis of speech in affective disorders. Archives of General Psychiatry 33, 1361 (1976). 985047 10.1001/archpsyc.1976.01770110089009

[21] Coppersmith G., Dredze M. & Harman C. Quantifying Mental Health Signals in Twitter. Proceedings of the Workshop on Computational Linguistics and Clinical Psychology: From Linguistic Signal to Clinical Reality (2014). doi:10.3115/v1/w14-3207

[22] Shing H.-C. Expert, crowdsourced, and machine assessment of suicide risk via online postings. Proceedings of the Fifth Workshop on Computational Linguistics and Clinical Psychology: From Keyboard to Clinic (2018). doi:10.18653/v1/w18-0603

[23] Tadesse M. M., Lin H., Xu B. & Yang L. Detection of depression-related posts in Reddit Social Media Forum. IEEE Access 7, 44883–44893 (2019).

[24] Zirikly A., Resnik P., Uzuner Ö. & Hollingshead K. CLPsych 2019 Shared Task: Predicting the Degree of Suicide Risk in Reddit Posts. Proceedings of the Sixth Workshop on Computational Linguistics and Clinical Psychology 24–33 (2019). doi:10.18653/v1/w19-3003

[25] De Choudhury M., Kiciman E., Dredze M., Coppersmith G. & Kumar M. Discovering shifts to suicidal ideation from mental health content in social media. Proceedings of the 2016 CHI Conference on Human Factors in Computing Systems (2016). doi:10.1145/2858036.2858207 PMC5659860 29082385

[26] Zhang T., Schoene A. M., Ji S. & Ananiadou S. Natural language processing applied to Mental Illness Detection: A Narrative Review. npj Digital Medicine 5, (2022). 10.1038/s41746-022-00589-7 PMC8993841 35396451

[27] He Q., Veldkamp B. P., Glas C. A. & de Vries T. Automated assessment of patients’ self-narratives for posttraumatic stress disorder screening using natural language processing and text mining. Assessment 24, 157–172 (2016). 26358713 10.1177/1073191115602551

[28] Baccianella S., Esuli A. & Sebastiani F. SentiWordNet 3.0: An Enhanced Lexical Resource for Sentiment Analysis and Opinion Mining. Proceedings of the Seventh International Conference on Language Resources and Evaluation (LREC’10) 2200–2204 (2010).

[29] Boyd R. L., Ashokkumar A., Seraj S. & Pennebaker J. W. The Development and Psychometric Properties of LIWC-22. Austin, TX: University of Texas at Austin (2022). doi:10.13140/RG.2.2.23890.43205

[30] De Choudhury M., Counts S., Horvitz E. J. & Hoff A. Characterizing and predicting postpartum depression from shared Facebook data. Proceedings of the 17th ACM conference on Computer supported cooperative work & social computing (2014). doi:10.1145/2531602.2531675

[31] Blei D. M., Ng A. Y. & Jordan M. I. Latent Dirichlet allocation. Journal of Machine Learning Research 3, 993–1022 (2003).

[32] Mikolov T., Sutskever I., Chen K., Corrado G. & Dean J. Distributed representations of words and phrases and their compositionality. Advances in Neural Information Processing Systems 26, 3111–3119 (2013).

[33] Lancaster C. A. Risk factors for depressive symptoms during pregnancy: A systematic review. American Journal of Obstetrics and Gynecology 202, 5–14 (2010). 20096252 10.1016/j.ajog.2009.09.007 PMC2919747

[34] Andersson S., Bathula D. R., Iliadis S. I., Walter M. & Skalkidou A. Predicting women with depressive symptoms postpartum with machine learning methods. Scientific Reports 11, (2021). 10.1038/s41598-021-86368-y PMC8041863 33846362

[35] Liu T. The relationship between text message sentiment and self-reported depression. Journal of Affective Disorders 302, 7–14 (2022). 34963643 10.1016/j.jad.2021.12.048 PMC8912980

[36] Edwards T. M. & Holtzman N. S. A meta-analysis of correlations between depression and first person singular pronoun use. Journal of Research in Personality 68, 63–68 (2017).

[37] Guntuku S. C., Yaden D. B., Kern M. L., Ungar L. H. & Eichstaedt J. C. Detecting depression and mental illness on social media: An integrative review. Current Opinion in Behavioral Sciences 18, 43–49 (2017).

[38] Morales M., Scherer S. & Levitan R. A cross-modal review of indicators for Depression Detection Systems. Proceedings of the Fourth Workshop on Computational Linguistics and Clinical Psychology –- From Linguistic Signal to Clinical Reality (2017). doi:10.18653/v1/w17-3101

[39] Tausczik Y. R. & Pennebaker J. W. The psychological meaning of words: LIWC and computerized text analysis methods. Journal of Language and Social Psychology 29, 24–54 (2010).

[40] Rude S., Gortner E.-M. & Pennebaker J. Language use of depressed and depression-vulnerable college students. Cognition & Emotion 18, 1121–1133 (2004).

[41] Friedman L. E., Gelaye B., Sanchez S. E. & Williams M. A. Association of Social Support and Antepartum Depression among pregnant women. Journal of Affective Disorders 264, 201–205 (2020). 32056751 10.1016/j.jad.2019.12.017

[42] Tølbøll K. B. Linguistic features in depression: a meta-analysis. Journal of Language Works - Sprogvidenskabeligt Studentertidsskrift 4, 39–59 (2019).

[43] Bernard J. D., Baddeley J. L., Rodriguez B. F. & Burke P. A. Depression, Language, and Affect: An Examination of the Influence of Baseline Depression and Affect Induction on Language. Journal of Language and Social Psychology 35, 317–326 (2015).

[44] Pennebaker J. W. Writing about emotional experiences as a therapeutic process. Psychological Science 8, 162–166 (1997).

[45] Watkins E. R. Constructive and unconstructive repetitive thought. Psychological Bulletin 134, 163–206 (2008). 18298268 10.1037/0033-2909.134.2.163 PMC2672052

[46] Loveys K., Crutchley P., Wyatt E. & Coppersmith G. Small but mighty: Affective micropatterns for quantifying mental health from social media language. Proceedings of the Fourth Workshop on Computational Linguistics and Clinical Psychology –- From Linguistic Signal to Clinical Reality (2017). doi:10.18653/v1/w17-3110

[47] Clark L. A. & Watson D. Tripartite model of anxiety and depression: Psychometric evidence and taxonomic implications. Journal of Abnormal Psychology 100, 316–336 (1991). 1918611 10.1037//0021-843x.100.3.316

[48] Scherer S., Lucas G. M., Gratch J., Rizzo A. S., & Morency L. P. (2015). Self-reported symptoms of depression and PTSD are associated with reduced vowel space in screening interviews. IEEE Transactions on Affective Computing, 7(1), 59–73.

[49] Kim J. A systematic review of the validity of screening depression through Facebook, Twitter, Instagram, and Snapchat. Journal of Affective Disorders 286, 360–369 (2021). 33691948 10.1016/j.jad.2020.08.091

[50] Kelley S. W. & Gillan C. M. Using language in social media posts to study the network dynamics of depression longitudinally. Nature Communications 13, (2022). 10.1038/s41467-022-28513-3 PMC8847554 35169166

[51] Mikolov T., Yih W. T. & Zweig G. Linguistic regularities in continuous space word representations. Proceedings of the 2013 Conference of the North American Chapter of the Association for Computational Linguistics: Human Language Technologies, 746–751 (2013).

[52] Krishnamurti T., Allen K., Hayani L., Rodriguez S. & Davis A. L. Identification of maternal depression risk from natural language collected in a Mobile health app. Procedia Computer Science 206, 132–140 (2022). 36712815 10.1016/j.procs.2022.09.092 PMC9879299

